# Embracing digital innovation in neuroscience: 2023 in review at NEUROCCINO

**DOI:** 10.1007/s00429-024-02768-6

**Published:** 2024-02-22

**Authors:** Eva Guzmán Chacón, Marcela Ovando-Tellez, Michel Thiebaut de Schotten, Stephanie J. Forkel

**Affiliations:** 1 Donders Institute for Brain Cognition Behaviour, Radboud University, Nijmegen, The Netherlands; 2 University Bordeaux, CNRS, CEA, IMN, UMR 5293, GIN, 33000 Bordeaux, France; 3 Brain Connectivity and Behaviour Laboratory, Paris, France; 4 Centre for Neuroimaging Sciences, Department of Neuroimaging, Institute of Psychiatry, Psychology and Neuroscience, King’s College London, London, UK; 5 Max Planck Institute for Psycholinguistics, Nijmegen, The Netherlands

**Keywords:** NEUROCCINO, MRI, Science communication, Clinical neuroanatomy, Neuroscience, Equity

As we reflect on the past year in neuroscience, it is evident that the field has reached an exciting juncture of discovery and innovation, significantly influenced by technological advancements. The traditional avenues of knowledge dissemination, including conferences and lab discussions, have been effectively augmented by digital platforms, fostering global scientific engagement. A prime example of this evolution is the non-profit Clinical Neuroanatomy Seminars, which transitioned online in 2016, well ahead of the digital curve. This bold move, initially aimed at broadening access to scientific seminars, became particularly crucial during the COVID-19 pandemic, transforming our initiative into a pivotal global scientific platform.

This innovative endeavour was recognised with the Education in Neuroimaging Award from the International Organization of Human Brain Mapping (OHBM), a testament to the channel's educational and scientific impact. The CNSeminar’s NEUROCCINO journal club, our flagship online series, has exemplified this digital leap. With a 38% increase in views, a 25% rise in watch hours, and a 17% growth in subscribers compared to the previous year, NEUROCCINO has become a beacon of knowledge and community in scientific communication in 2023.

Presenting at NEUROCCINO is open to everyone, with a particular emphasis on the involvement of early career researchers. The meeting, held for 30 min every Monday morning, accumulated 17 h of scientific discussions in 2023. With a total of 576 h watched, this indicates that for every hour of content produced, there are 34 h invested in viewership. The channel has attracted audiences from 39 countries, with significant engagement metrics indicating a strong resonance with our viewers (Fig. 1).

**Fig. 1:**
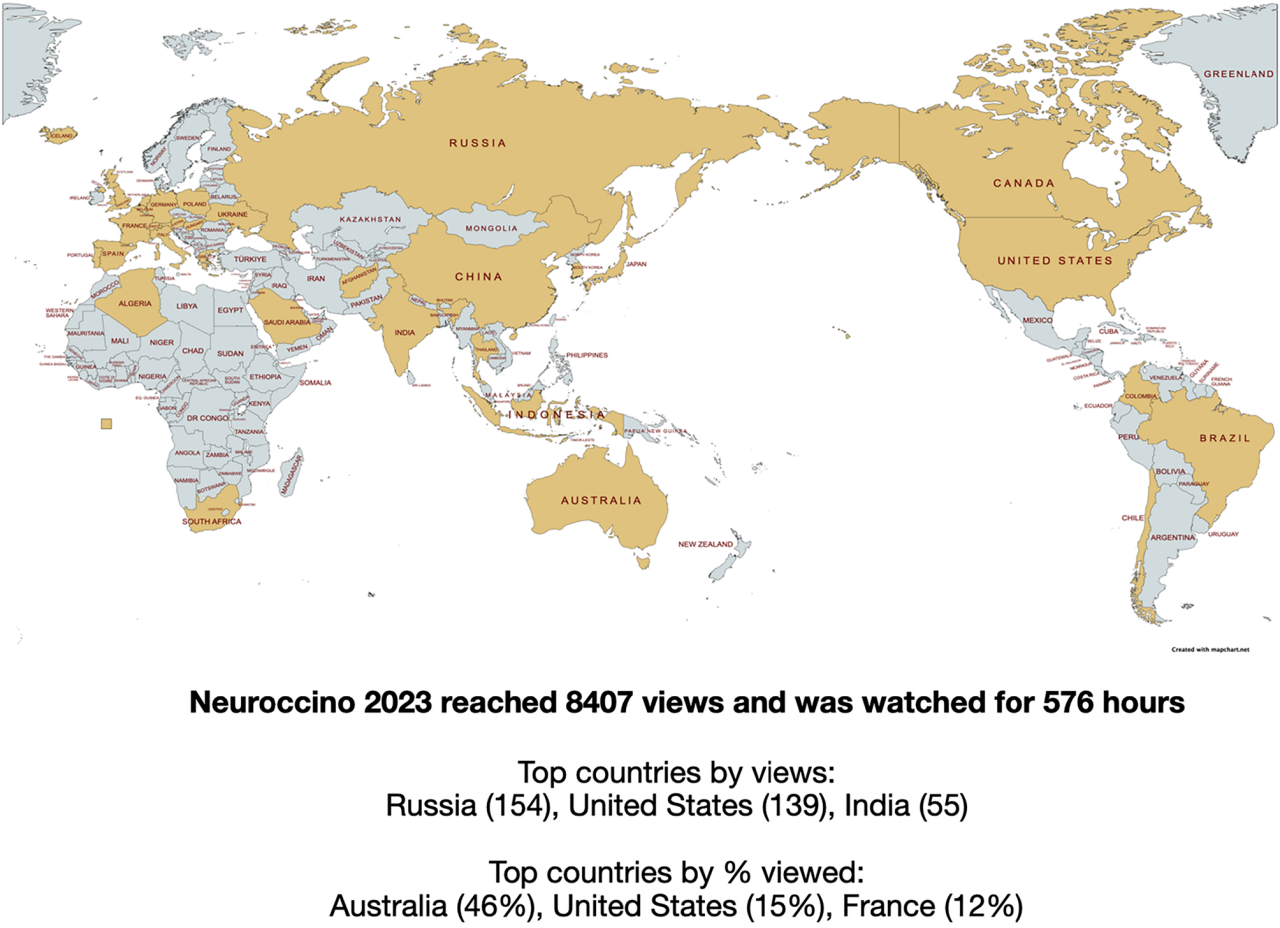
Global reach of the NEUROCCINO online journal club (created with mapchart.net)

The thematic direction of the presentations emerges organically from the contributors, independent of a predetermined scientific directive. We have noticed annual thematic trends, and 2023 is no exception, with our exploration of cognition notably skewed towards language (31% of journal clubs) and an additional 4 sessions hinting at potential implications for the field of language research. This year, we facilitated 35 Journal Clubs featuring presentations by Master’s students (3%), PhD candidates (31.5%), Postdoctoral researchers (34%), and Principal Investigators (31.5%). In total, 17 individuals, comprising 9 women and 8 men, participated in the presentations. Despite the gender balance among volunteers, women delivered nearly twice as many presentations as men, with a count of 22 versus 12 (1 session was not counted as it was a co-hosted event). The scientific discoveries, methodological and technological innovations, and debates of 2023 are described in detail below.

## Scientific controversies and debates

The year 2023 witnessed significant scientific controversies and debates. The discussions around localizationism and its alternatives, such as the ‘Wernicke conundrum’ and the ‘call to embrace anti-localizationism’, have been particularly engaging. These debates underline the ongoing evolution of our fundamental understanding of brain structure and function.

The debates on the framework of localizationism have reappeared in many journal clubs. NEUROCCINO inaugurated the year with an in-depth analysis of the ‘Wernicke conundrum’—a series of publications in the Journal Brain (YouTube ID: H3J4FH1Kpsw; Mesulam et al. 2015, 2023; Matchin et al. 2022, 2023) discussing the anatomical foundation of comprehension and repetition deficits with localised anterior temporal pole lesions versus a disconnection framework (x6K6KipyBjI; Forkel et al. 2020; Janssen et al. 2023). This year was rounded off with a discussion on a manuscript calling to ‘embrace anti-localizationism’ (vZPk-teJxHE; Noble et al. 2023) as ‘partial holism’. The ensuing debate primarily concerned the need for synchronicity between methods and theory when adopting a framework. In addition, there was advocacy for not completely discarding any framework but rather recognising its complementary value (vZPk-teJxHE; Noble et al. 2023). A historical perspective was given by revisiting Hewlett Jackson’s 19th-century theories highlighting that these ideas are far from novel. Jackson questioned the dominant notion of hemispheric localizationism, proposing a ‘mirror-like’ functionality in the brain, suggesting that both hemispheres contain and process words but in different manners (_UU-LkgCfro; Jackson 1874). This debate was embedded into a clinical setting demonstrating limitations of clinical assessments due to the discourse around varying definitions and evaluations of aphasia (X9GqM4DPufo; Castro et al. 2023), which largely hinge on broader yet contested frameworks like localizationism. Our inspection of brain function steered away from the cortico-centric view with a presentation on the thalamus’ integrative role in language processing (2JrHX0j_COE; Shine et al. 2023) and an exploration of the cerebellum’s predictive power in non-motor function (W7Bb3S4sCnM; Sokolov et al. 2017).

Another discussion delved into the definition of entropy as a measure of the subjacent complexity inherent to any measure. Its significance in neuroscience and a mathematical demonstration were discussed, emphasising the need for a deeper understanding of entropy for functional MRI studies. The presentation also explored the challenges in quantifying and interpreting entropy due to subjectivity and measurement limitations (NeZLE3NPMg0; Fagerholm et al. 2023).

Groundbreaking anatomical discoveries were also sprinkled across the year, including the description of an additional meningeal layer around the brain (TGZrO6GrB2I; Møllgård et al. 2023). This discovery was rapidly embraced and integrated into neuroanatomy education, albeit debated since the 1970s (Nabeshima et al. 1975; Krisch et al. 1983; Balin et al. 1986; Haines 1991). The year 2023 ushered in the era of new atlases, with the Human Brain Project’s legacy EBRAINS (UG8pT94s130; Naddaf 2023) and a new method for creating a detailed cell census of the human brain’s frontal lobe, integrating high-resolution imaging techniques to map cellular structures within a whole-brain reference space (OnTgvienNBs; Costantini et al. 2023). A revived discussion surfaced following a publication asserting the fundamental influence of brain geometry (shape) on the brain’s functional dynamics (pSKYGuXXb0s; Pang et al. 2023). While the originality of this assertion faced scepticism from various groups, a team employing open-source code scrutinised these claims. Their analysis revealed that a spherical model of the brain possesses equivalent predictive capabilities as its folded counterpart, thereby challenging the paper’s core assertion (https://colab.research.google.com/drive/1MrkHAenYXn7EXrwKcwVyMT6KbcjrXHMm).

As these debates continue, they offer valuable insights into the intricacies of scientific inquiry and the importance of open and respectful discourse in pushing the boundaries of knowledge.

## Ethical considerations

The impact of technology on neuroscience was another key theme and watershed moment in 2023. The emergence of AI tools, like ChatGPT, has sparked meaningful conversations about the reliability of AI-generated content and the need for ethical considerations in AI usage, where technological advancements increasingly intersect with ethical deliberations (Sesack and Thiebaut de Schotten 2023; Ruffle, Foulon, Nachev, 2023). NEUROCCINO discussed tools like ChatGPT, addressing the reliability of AI content, its impact on critical thinking, and the need for regulation (5kAFlBty4Sk). The topic of trustworthiness in machine learning models and their vulnerability to data manipulation was debated, advocating for the publication of raw data to enable the replication and validation of results (ArcHsUP7e5E; Rosenblatt et al. 2023). The implications of these findings underscored the critical need for rigorous data validation and replication in research, as well as the development of strategies to prevent data manipulation and enhance the trustworthiness of machine learning models. The prominence of AI was also evidenced in presentations on technologies such as transfer learning in neuroscience (NFsL99Ey5Lk; Ardalan and Subbian 2022) and inferential analyses where the performance of different algorithms is used to infer the brain's inner model on a given task (wZEYFwdvgdE; Janini et al. 2022).

Going into the new year and continuously learning about new AI developments, we will likely revisit some of these debates and innovations with a more educated mind.

## Emerging tools and technology

Our focus on innovative tools and methodologies has also been evident. Discussions on transfer learning, semantic reconstruction from brain recordings, and new techniques such as hyperalignment (Jiahui et al. 2023) and CEBRA (Schneider et al. 2023) (further considered below) have highlighted the potential of technology in advancing our understanding of the brain.

An emerging trend focuses on interindividual differences and novel tools to map them. We discussed a new method to estimate individual brain states at single time points using resting state, its effectiveness in post-stroke recovery, and its limitations, including reliance on atlas-based estimation and signal simplification (fjGI2pXL71M; Peng et al. 2023). Despite its limitations, this method holds significant implications for neurological research and clinical applications, particularly in understanding individual brain organisation and post-stroke recovery, underscoring the need for ongoing research and developments in clinical neuroimaging.

Decoding individual brain signals, often referred to as ‘mind reading’ in the lay media, was also exemplified by the ‘Pink Floyd study’, where neural activity was recorded from 29 people with epilepsy with intracranial electrodes who listened to Another Brick in the Wall (Bellier et al. 2023). Using independent component analysis (ICA), meaningful components of the neural activity related to the music, such as vocals and instruments, were extracted. The discussion highlighted the need for well-defined data pools, the lack of generalisability given the invasive nature of the study, and the lack of control for song familiarity (9 × 23fk_fHoM; Bellier et al. 2023). Another study also delved into the details of Direct Electrical Stimulation Mapping (DES) in neurosurgery, highlighting its invasive yet crucial role in localising functional boundaries around tumours to prevent harm to the language system (leEddoDBihk; Collée et al. 2023). Two studies investigated non-invasive alternatives using temporal interference (wNEoiQbRSes; Violante et al. 2023) and fMRI for stimulation and developing brain-computer interfaces to decode language and semantic information. The latter revealed that multiple brain regions work together to encode language representations, thereby reconstructing semantic information from individual brain regions (UnHjhHS9ACo; Tang et al. 2023).

Methodological advances were also prominent this year. Discussing a review of studies using transfer learning, focussing on improving predictions and classification accuracy, highlighted the limitations, such as the lack of publicly available 3D convolutional neural networks and the difficulty of applying transfer learning to neuroimaging data. Questions were raised about the applicability of transfer learning to smaller data sets and its effectiveness in classifying different disorders, especially those that are less well defined, such as psychiatric disorders (NFsL99Ey5Lk; Ardalan and Subbian 2022).

A novel method known as hyperalignment was also introduced. This technique uses connectivity measures to create category-selective maps, traditionally accomplished with functional localisers, to map specific brain regions (yNfKoMa81W8; Jiahui et al. 2023). A new dimensionality reduction technique, CEBRA, was discussed, which builds a joint representation space for neural recordings and behavioural labels, addressing some limitations of existing methods such as Principle Component Analysis (PCA) and its non-linear counterpart Uniform Manifold Approximation and Projection (UMAP) (Schneider et al. 2023). At the same time, a discussion unfolded about the dimensionality of the models used and the inclusion of time information in the algorithms (h55la-1lrzY).

As we progress, these tools will be subject to greater application and scrutiny, ultimately needing to withstand the test of time.

## Conclusion

As we look towards the scientific landscape in the upcoming year, the field of neuroscience stands poised for groundbreaking advancements. The dynamic changes triggered by technology, particularly in the realm of digital communication, have catalysed a remarkable shift in how scientific knowledge is disseminated and discussed. We are excited about the prospects of further integrating digital innovations into neuroscience. Our commitment to accessible, inclusive, and engaging scientific discourse remains steadfast. We are poised to continue leveraging technology to break down barriers, foster global collaborations, and help drive the field of neuroscience towards new frontiers of discovery and understanding. Join us as we embark on another year of exploration and learning at the intersection of neuroscience and digital innovation. Your participation, whether as a presenter, viewer, or contributor, makes NEUROCCINO a vibrant and dynamic platform for scientific exchange. Together, let us shape the future of neuroscience—one journal club at a time.

## Data Availability

The video transcript dataset was generated by batch downloading the Neuroccino playlist using Media Downloader (github.com/mhogomchungu/media-downloader), transcribed using the Whisper model, and summarised with Summascript (portfoliopals.net/summascript) and ChatGPT4. The code and video summaries are available at github.com/eva-gc/Neuroccino-2023. The YouTube Analytics were exported from YouTubeStudio and are available through the links above. The entire Neuroccino 2023 playlist is available at https://youtube.com/playlist?list=PLg2e4R8Sdhpe8J_sITZc6XYmJENuVJJkD&si=H4qXam7rzRnml7_Q with details available from www.stephanieforkel.com/cnseminars.
